# Optimization of pulsed electric field conditions for sugar extraction from carrots

**DOI:** 10.1002/fsn3.1490

**Published:** 2020-03-04

**Authors:** Samere Dastangoo, Mohammad Taghi Hamed Mosavian, Samira Yeganehzad

**Affiliations:** ^1^ Department of Chemical Engineering Faculty of Engineering Ferdowsi University of Mashhad Mashhad Iran; ^2^ Food Processing Department Research Institute of Food Science and Technology (RIFST) Mashhad Iran

**Keywords:** carrot, optimization, pulsed electric field, sugar extraction

## Abstract

Physical destruction and thermal treatment are pretreatment methods used to destroy cell membranes and facilitate the release of solute extraction. In this paper, sugar extraction from carrots under different pulsed electric field conditions (field strengths of 250, 750, and 1,250 V/cm, pulse numbers of 10, 45, and 80, and pulse frequency of 1 Hz) and simultaneous thermal treatments (at 20, 45, and 70°C) were studied based on full factorial design experiments with 27 runs. Carrot slices treated with PEF were suspended in water at the desired temperature and liquid‐to‐solid weight ratio of *L/S* = 2. Immediately after the PEF treatment, a significant increase in solute extraction was observed due to the permeability of cell membrane that could lead to the enhancement of solute convection on the surface of the tissue. Optimum extraction parameters were obtained as follows: PEF with the field intensity of 750 V/cm, 10 pulses, and temperature of 45°C.

## Graphical abstract

Novel pretreatment methods have been applied to improve efficiency in separation processes such as extraction and diffusion. It has been shown that pulsed electric field is a very effective method in permeabilizing of biological cell membranes that allows aqueous penetration of valuable materials from the plant tissue matrix at ambient temperature, which leads to energy and time saving. This paper studies the effects of PEF and thermal treatments simultaneously on the texture of carrot tissue. The observed effects may be remarkable from an industrial point of view for growth of the PEF‐assisted sugar extraction.

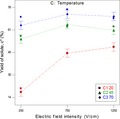

## 1 INTRODUCTION

Sugars exist in most of the plant tissues, but only sugar beet and sugar cane are significant sources of sugar for optimal extraction in the industry. In general, carrot with 4.7% of total sugar is placed after sugar beet, sugar cane, and sweet corn, respectively, and could be considered as a source of sugar (Dubounet, 2011).

Extraction as the first step of plant tissue study plays an important role in the final result and the following processes. The extraction process is influenced by factors such as matrix properties of the plant, solvent, temperature, pressure, and time (Azmir et al., 2013). The complete extraction of intracellular components in a solid–liquid extraction process involves the denaturation of cell membranes in the solid phase. In this way, sugar is extracted by countercurrent water at the high temperature of liquid phase within 70–74°C during the extracting times of 1–1.5 hr, which leads to the disintegration of cell membranes. The disadvantage of these processes is the decomposition of the inner chemical structure of cell walls by hydrolytic reactions. At such elevated temperatures, the amount of colloidal substances such as proteins, pectin, and other polluting materials entering the raw juice would be increased, resulting in low‐purity raw juice which is very harmful for evaporation units (Lopez, Puertolas, Condon, Raso, & Alvarez, 2009; Sotudeh‐Gharebagh, Shamekhi, Mostoufi, & Norouzi, 2009; Van der Poel, 1998). Nonconventional methods, which are more environmentally friendly, reduce operational time, and have better yield and quality of extract, have been developed during the last 50 years (Azmir et al., 2013). To enhance the overall yield and selectivity of components from plant materials, ultrasound (Karki et al., 2010; Mason, Paniwnyk, & Lorimer, 1996), pulsed electric field (A B Jemai & Vorobiev, 2006; Toepfl, Mathys, Heinz, & Knorr, 2006), enzyme digestion (Wang, Yang, & Wei, 2010), microwave heating (Li, Jia, Wei, & Liu, 2012), supercritical fluids (Cardenas‐Toro et al., 2014), and accelerated solvents (Nayak et al., 2015) have been studied as nonconventional methods. PEF^1^ is a nonthermal extraction method to improve the membrane that allows cold or hot aqueous penetration of valuable materials from the plant matrix. During recent years, several studies have been conducted about the effects of PEF on the extraction of soluble materials from plant tissues (El Belghiti & Vorobiev, 2004; Jemai & Vorobiev, 2003; El‐Belghiti, Rabhi, & Vorobiev, 2005; Fincan, DeVito, & Dejmek, 2004; Jemai & Vorobiev, 2002; Corrales, Toepfl, Butz, Knorr, & Tauscher, 2008). When the sample is exposed to an electric field of sufficiently high intensity, the cell membrane can be irreversibly degraded (El‐Belghiti & Vorobiev, 2005). The destruction of a biological cell (plant or animal) with a high‐intensity electric field in the form of very short pulses causes the formation of pores on the cell membrane that can be either temporary or permanent. This phenomenon which is known as electroporation enhances the permeability of the treated cells, eventually leading to the breakdown of cell membranes (Mandal, Mandal, & Das, 2015). Applying the PEF processing at moderate electric field intensity (*E* < 100 V/cm) and room temperature, or moderate thermal treatment (*T* < 50°C) without any electric field, requires a long treatment time (PEF or thermal) and, subsequently, consumes a great deal of energy. Applying PEF and thermal treatment simultaneously has a strong effect on tissue damage (Lebovka, Praporscic, Ghnimi, & Vorobiev, 2005; Lebovka, Shynkaryk, El‐Belghiti, Benjelloun, & Vorobiev, 2007). This effect can lead to structural transitions occurring inside membranes at high temperatures (Zimmermann, 1986). The kinetics of these processes depends on the parameters of PEF (field intensity, number of pulses, and pulse duration), external solute transfer conditions (stirring of solution), and the ratio of liquid‐to‐solid, and effects of sample size (El Belghiti & Vorobiev, 2004).

This work aims to apply full factorial design (FFD) methodology to study the simultaneous effect of PEF parameters (field intensity and the number of pulses) and temperature on sugar extraction yield and the disintegration index of carrot slices to obtain the optimal conditions of PEF and thermal treatments. Compared with other published findings, the data analysis and optimization process of these two factors resulted from the deficiency of a one‐variable‐at‐a‐time approach. The conventional method of maintaining other factors involved in a study at a constant level and studying each factor separately does not include the interaction effects of all the factors and is time‐consuming as well. Therefore, a statistical experimental design minimizes the above‐mentioned difficulties by studying all the significant factors simultaneously (Elibol, 2002). The factorial design methodology is an important tool for data analysis and finding optimal process conditions. Using this method leads to a more efficient experimental design that avoids misleading conclusions (Gheshlaghi, Scharer, Moo‐Young, & Douglas, 2008).

## 2 MATERIALS AND METHODS

### 2.1 Sample preparation

Carrot samples used in this study were supplied from the special carrot farm with known species, in Mashhad, wrapped in sealed bags, and stored for 2–3 days in the refrigerator at 4 ± 2°C until experimental testing. The carrots were washed, and all the wastes were cleaned from the surface. Upper and lower parts of each carrot (approximately 2 cm) were removed. Then, the carrots were cut into disk pieces of 1 ± 0.1 cm in thickness, 3 ± 0.2 cm in diameter, and 10 ± 0.5 g in weight for the experiments. For each experiment, 10 pieces of carrot with the total weight of 100 ± 0.50 g were weighed by a balance (model FX3000, AND, Japan).

### 2.2 PEF pretreatment

Carrot disk samples were placed inside a Plexiglas treatment chamber with dimensions of 4 × 10×10 cm^3^ between two stainless steel electrodes with 4 cm of gap and area of 10 × 10 cm^2^. Then, tap water (0.05 S/m, 20°C) was added to the treatment chamber at the room temperature, so that the carrot slices would be immersed in water completely. The liquid‐to‐solid weight ratio was fixed at the value of 2 (*L/S* = 2). The PEF generator (1,750 V‐20 A, Food Tech, Iran) could create electrical current of 1,750 V/cm with exponential decay wave pulses. Electrical current from an AC power supply (220–240 V 50 Hz) was transferred to a DC power supply. Then, electrical energy was transferred to a series of capacitances by a linear current, and the energy stored in the capacitors was discharged to the electrodes and treatment chamber by a pulse key. The energy of each pulse (Q) used in PEF treatment during exponential decay was given by Equation (1) (Maskooki & Eshtiaghi, 2012):

(1) $$Q=\frac{V^{2}\times C}{2}$$

where *V* is the peak value of decaying voltage (volt) in the sample and *C* is the capacity of the condenser (*F*). The temperature changes (Δ*T*) in the treatment chamber due to dissipation electrical energy in the chamber (nQ) during PEF treatment were calculated using the following equation:

(2) $${\text{mc}}_{p}\Delta T=\text{nQ}$$

where *c_p_* and m are the specific heat and mass of samples, respectively. The relation between cell disintegration index (*Z*) and energy input under different PEF treatment conditions was expressed as the equation below:

(3) $$E_{\text{eff}}=Z/\text{nQ}$$

where *E*_eff_ is energy efficiency, *Z* cell disintegration index, and nQ is total energy input. This index showed effective cell disintegration per unit energy input by PEF treatment. This index showed effective cell disintegration per unit energy input by PEF treatment. PEF parameters included the electric field intensities of 250, 750, and 1,250 V/cm as well as different numbers of monopolar pulses of 10, 45, and 80. The pulse frequency was kept at constant 1 Hz. The juice elevated temperature was negligible (about 1°C).

### 2.3 Thermal treatment

Immediately after PEF pretreatment, the samples were immersed in the juice that had previously reached the desired temperature (20–45–70°C) using a magnetic agitator (0–310°C, 0–1,500 rpm, IKA, RCT basic, Germany) according to the experimental design. In all the experiments, the solution was stirred at 250 rpm in a closed cylindrical device to prevent evaporation effects during the extraction process.

### 2.4 Determination of extraction yield (c*)

To study the extraction kinetics, the solute concentration (Brix, g soluble 100 g^−1^ juice) was measured by a digital refractometer (RX‐5000α, Atago, Japan) every 15 min up to 4 hr during the extraction process. The mathematical model used in this study was the one proposed by So and MacDonald (1986), which involved two first‐order kinetics simultaneously. The first stage was washing which was related to the transfer of solute by convection and occured quickly; the second stage was diffusion related to the prolonged solute transfer by diffusion from the intracellular plant tissue matrix. This model can be described by Equation (4) (El Belghiti & Vorobiev, 2004; El‐Belghiti, Rabhi, & Vorobiev, 2007):

(4) $$c^{*}=c_{w}^{*}\left(1-e^{-k_{w}t}\right)+c_{d}^{*}\left(1-e^{-k_{d}t}\right)$$

where c* can be described according to Equation (5):

(5) $$c^{*}=c/c_{\infty }$$

where *c* is the actual solute concentration in the solution, *c_∞_* is the equilibrium solute concentration, $c_{\infty }≅c_{0}/\left(n+1\right)$, *c*_0_ is the concentration of juice in fresh plant tissue in g[soluble]/100 g [juice], *n* is the liquid‐to‐solid ratio (*L/S* = 2), *L* is the extract volume in m^3^, *S* is the solid volume in m^3^, $c_{w}^{*}$ is the *c_w_/c_∞_* ratio, *c_w_* is the equilibrium solute concentration due to the washing stage alone, $c_{d}^{*}$ is the *c_d_/c_∞_* ratio, *c_d_* is the equilibrium solute concentration due to the diffusion stage alone, *k_w_* is the kinetic coefficient for the washing stage in min^−1^, *k_d_* is the kinetic coefficient for the diffusion stage of extraction in min^−1^, and *t* is time in min.

### 2.5 Determination of disintegration index (Z)

At the end of each experiment, the electrical conductivity of each piece was determined using a conductivity meter (model IDSC004) before and after PEF and thermal treatment by measuring the electrical current passing through the sample connected to a 12 V DC source by the following equation (Sakr & Liu, 2014):

(6) $$\sigma =\frac{L}{A}\times \frac{I}{V}$$

where *L* is the gap between two electrodes in m, *A* is the cross‐sectional area of carrot samples in the cell in m^2^, *I* is the alternative current passing through the material in *A*, and *V* is the voltage across the material in *V* that is constant. The degree of tissue damage was estimated from the electrical conductivity disintegration index *Z* (Angersbach, Heinz, & Knorr, 2002; Lebovka, Shynkaryk, & Vorobiev, 2007b, 2007c):

(7) $$Z=\left(\sigma -\sigma_{i}\right)/\left(\sigma_{d}-\sigma_{i}\right)$$

where *σ* is the measured electrical conductivity in S/m, and the subscripts *i* and *d* refer to the conductivities of intact and completely damaged tissues, respectively. The maximally damaged carrot tissues *σ_d_* were obtained by measuring the electrical conductivity of freeze‐thawed tissue (Lebovka, Shynkaryk, El‐Belghiti, et al., 2007; Lebovka et al., 2007b, 2007c).

### 2.6 Experimental design and data analysis

To develop a mathematical model for extraction yield and disintegration index as dependent variables in terms of predictor variables (field intensity, number of pulses, and temperature), a sequential approach using the design of experiments was carried out.

Each variable was coded according to Equation (8):

(8) $$x_{i}=\frac{X_{i}-\left(X_{i,\text{high}}+X_{i,\text{low}}\right)/2}{(X_{i,\text{high}}-X_{i,\text{low}})/2}$$

where *x_i_* is the coded value of variable *X_i_*, and *X_i,_*_low_ and *X_i,_*_high_ are the values of the variable at low and high levels, respectively (Gheshlaghi et al., 2008; Montgomery & Design, 2001).

### 2.7 Modeling and optimization by full factorial design (FFD)

Full factorial design is a strong candidate in which all the possible replacements of all factors and possible interactions within factors are tested, and each test is replicated at least twice and the results are analyzed using ANOVA^2^ (Gottipati & Mishra, 2010; Morrison, Block, Strickland, Collier, & Peterson, 2008). Thus, this method has greater precision in estimating the overall main factor effects and their interactions (Gottipati & Mishra, 2010).

If the combinations of k factors are applied at three levels, a factorial design will consist of 3^k^ experiments (Montgomery, 1982). In the present study, a three‐factor three‐level FFD with two replicates at each factor combination (3^3^ runs) was carried out to develop a mathematical model for extraction yield and disintegration index. The levels of the three variables for the FFD are indicated in Table 1. The low and high levels of the factors were selected based on some preliminary experiments.

**Table 1:** Levels of independent variables in FFD

Independent variable	Coded symbol	Range and level		
		−1	0	+1
Electric field intensity (V/cm)	A	250	750	1,250
Number of pulses	B	10	45	80
Temperature	C	20	45	70

After modeling the responses by FFD, this method was developed for the optimization of variables affecting the two investigated responses.

### 2.8 Statistical analysis

The data analysis and graphics as well as optimization process were performed using the experimental design software Design–Expert (version 6.0.4). The goodness of the fitted model was evaluated by the coefficient of determination, *R* ^2^. Furthermore, an adequate precision statistic was used to measure the signal‐to‐noise ratio. A ratio of greater than 4 is desirable. The normality of the errors was checked by the normal probability plot of the residuals. To detect the correlation between the residuals, the plot of residuals in time sequence was checked so that the residuals are structureless in the correct model (Montgomery, 1982) (not shown). The significance of a fitted model and independent variables was determined using ANOVA with Fisher's test (*P*‐value < 0.05).

## 3 RESULTS AND DISCUSSION

Electric field intensity and the number of pulses are the main characteristics of a PEF protocol that influence extraction yield and disintegration index. The purpose of the first step of experiments (FFD) was to investigate the contribution of each factor in responses and determine their interaction effect as well. The second step was performed for finding the best conditions of the variables to achieve high performance of responses. The levels and ranges of the three factors for FFD are given in Table 1. This value was based on preliminary investigations. The experimental matrix for the 3^n^ factorial design (n factors, each run at three levels) for extraction yield and disintegration index is summarized in Table 2. The results were analyzed in Design–Expert software, and the main effects and interactions between the factors were determined.

**Table 2:** Design matrix and results of the 3^3^ full factorial design for the responses of solute extraction yield and disintegration index based on the extraction time of 240 min

Std.	Variable levels			Responses	
	A (V/cm)	B	C (°C)	c* (%)	Z
1	−1	−1	−1	16.07	0.0289
2	0	−1	−1	44.81	0.3186
3	+1	−1	−1	47.40	0.3204
4	−1	0	−1	20.18	0.0843
5	0	0	−1	44.46	0.2573
6	+1	0	−1	48.80	0.3441
7	−1	+1	−1	16.89	0.0604
8	0	+1	−1	44.34	0.3523
9	+1	+1	−1	50.21	0.2976
10	−1	−1	0	53.26	0.3067
11	0	−1	0	63.82	0.4274
12	+1	−1	0	60.07	0.5227
13	−1	0	0	56.31	0.4394
14	0	0	0	62.72	0.4077
15	+1	0	0	62.88	0.4712
16	−1	+1	0	53.26	0.4487
17	0	+1	0	66.40	0.4615
18	+1	+1	0	57.95	0.3494
19	−1	−1	+1	58.89	0.255
20	0	−1	+1	64.99	0.366
21	+1	−1	+1	75.32	0.5141
22	−1	0	+1	69.45	0.4444
23	0	0	+1	74.61	0.478
24	+1	0	+1	68.51	0.6983
25	−1	+1	+1	62.88	0.3506
26	0	+1	+1	74.61	0.4735
27	+1	+1	+1	65.70	0.5097

### 3.1 Kinetic of sugar extraction process

Table 3 shows the effect of electric field intensity at 45 pulses on the kinetic of solute extraction at 20, 45, and 70°C. Increasing both temperature and PEF pretreatment increased the extraction kinetics. By studying these charts, the effect of temperature on solute extraction kinetics was determined. For example, to achieve the extraction yield of 40% at 20°C and PEF pretreatment at 750 V/cm, the required time was 210 min, while 75 min and 90 min were required to achieve the same output at 45 and 70°C, respectively.

**Table 3:** Yield of solute during extraction process under number of pulses *n* = 45 from carrot slices treated with different electric field intensity at temperatures (a) *T* = 20°C, (b) *T* = 45°C, and (c) *T* = 70°C

Time (min)	c*, 20°C				c*, 45°C				c*, 70°C			
	250 V/cm	750 V/cm	1,250 V/cm	Untreated	250 V/cm	750 V/cm	1,250 V/cm	Untreated	250 V/cm	750 V/cm	1,250 V/cm	Untreated
0	0.00	0.00	0.00	0.00	0.00	0.00	0.00	0.00	0.00	0.00	0.00	0.00
15	4.69	9.27	8.45	1.17	5.87	8.06	13.00	4.00	10.00	11.00	12.00	8.00
30	7.51	17.13	17.36	1.29	11.73	20.10	24.64	8.00	20.88	25.57	21.59	18.00
45	9.15	20.76	22.52	1.82	17.36	28.16	31.79	12.00	30.62	35.66	29.09	23.00
60	10.21	23.35	26.04	1.99	21.59	32.85	36.37	15.50	35.66	40.83	35.43	28.00
75	11.26	26.63	29.09	2.17	25.81	37.07	40.59	19.30	39.77	46.46	40.12	32.00
90	12.67	28.98	31.91	2.29	29.80	40.98	44.46	23.00	43.64	49.98	45.05	36.00
105	13.61	31.21	34.26	2.23	33.32	43.95	47.40	26.51	46.93	53.50	48.33	39.00
120	14.20	33.20	36.13	2.46	36.37	46.46	49.16	30.50	49.74	56.31	51.38	41.35
135	14.90	35.08	38.24	2.46	39.42	49.04	51.62	33.90	53.26	59.13	53.50	43.99
150	15.95	36.95	39.65	2.52	42.23	51.46	53.97	37.00	55.73	61.94	56.66	46.87
165	16.66	37.42	41.30	2.52	44.58	53.97	55.37	39.54	57.13	64.52	58.66	49.39
180	17.83	39.18	42.70	2.93	46.69	55.92	56.55	42.00	60.65	66.17	60.53	51.00
195	18.54	39.89	44.11	2.64	48.80	57.33	58.19	44.23	62.29	68.04	62.88	53.00
210	18.77	41.30	45.28	2.70	50.92	58.58	59.48	46.22	64.05	69.69	64.17	54.00
225	19.01	42.47	46.46	2.87	52.79	60.07	60.30	47.98	66.17	72.03	65.70	56.00
240	19.59	43.64	47.63	2.99	54.67	61.79	61.71	49.62	67.46	73.21	67.10	57.00
255	20.18	44.46	48.80	2.76	56.31	62.73	62.88	51.00	69.45	74.61	68.51	59.00

Comparison between untreated samples and PEF pretreated samples was shown that at the temperature of 20°C after 255 min, the yield of extraction was not considerable for untreated samples, and the trivial extraction yield of 3% was due to the mechanical destruction of the carrot's tissue cells. By increasing the electric field intensity, the extraction yield due to the degradation of cell membranes significantly increased. At higher temperatures of 45 and 70°C, there was a significant difference (*p* < .05) by a confidence level of 95% between the untreated and PEF pretreated samples. The extraction yield was increased significantly by increasing the electric field intensity and, subsequently, the degradation effect of tissue at 45°C, although the difference in higher electric field intensity was less. The extraction yield was declined at 70°C by increasing the electric field intensity in the range of 750–1250 V/cm. This effect can be related to the accumulation of solute materials within the large constituent cells in the degraded tissue caused by a high electric field and high temperature. These results were in accordance with other studies about the synergistic effect of electrical and thermal treatments done on sugar beet tissue (El‐Belghiti, Rabhi, & Vorobiev, 2005; Lebovka, Shynkaryk, El‐Belghiti, et al., 2007; Maskooki & Eshtiaghi, 2012).

### 3.2 Solute extraction yields (c*)

The main effects and the interacting factors affecting the extraction yield were determined by ANOVA. For extraction yield, the main effects A and C and the interaction AC were of higher statistical significance, with a 95% confidence level. The model *F*‐value of 29.10 indicated that the model was significant. Based on F‐ratio and *p*‐value, statistically insignificant factors were discarded. Afterward, the following 2FI^3^ refined model was statistically considered in explaining the extraction yield:

(9) $$c^{*}=54.99-9.75A+5.09A^{2}-17.97C+4.64C^{2}-9.56AC+2.43A^{2}C+4.40AC^{2}-0.41A^{2}C^{2}$$

Comparing the relative weights of various terms in the model signified that temperature (C) had a very important effect on the extraction yield. To ensure an appropriate model, the *R* ^2^ value (0.9699) of the refined model was compared and deemed in agreement with the adjusted coefficient of determination, $R_{\left(\text{adj}\right)}^{2}$ value (0.9565).

**Figure 1:**
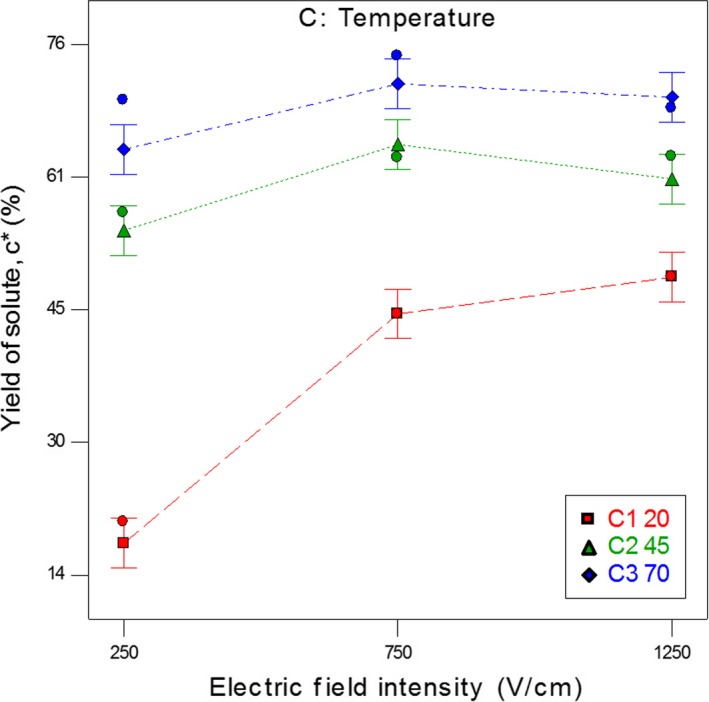
Interaction effects of electric field intensity and temperature on the solute extraction yield of carrot at *n* = 45 pulses after 255 min of thermal treatment

Figure 1 shows the interaction effects of electric field intensity and temperature on the solute extraction yield of carrot after PEF treatments at 45 pulses. From Equation 9, it is evident that the number of pulses and its interactions with electric field intensity (AB), as well as temperature (BC), are not significant. According to Angersbach, Heinz, and Knorr (2000), when the pulse width which is in the microsecond range (for the cell in potato tissue, *τ* = 0.7 µs; in apple tissue, *τ* = 1.4 µs; and in plant cell culture suspension, *τ* = 0.45 µs) became several times larger than the specific disintegration time (*τ*), the critical breakdown transmembrane potential that is the main cause of the membrane destruction did not depend on pulse duration and number of pulses (Angersbach et al., 2000). Therefore, there was no significant difference by varying the number of pulses between 10 and 80 at the 95% confidence level.

According to Figure 1, at 20°C and the electric field intensity of 250 V/cm, only 17% of solute materials were extracted. As the energy consumption increased during PEF pretreatment and electric field intensity increased from 250 V/cm to 1,250 V/cm, the permeable cells and, subsequently, yield extraction enhanced up to 750 V/cm. However, by increasing the field intensity to higher than 750 V/cm, no significant difference was observed in solute extraction yield at the 95% confidence level. It can be expected that this amount of energy is enough for the maximum permeability of the cells. Also, with increasing temperature in the range of 20 to 70°C, the solute extraction yield increased.

### 3.3 Cell disintegration index

The cell disintegration index of each run was calculated, and the responding values of FFD were utilized for ANOVA. The *F*‐value of 22.57 for the model implied that the model was significant. Moreover, the main effects of temperature and electric field, as well as the interaction effect, highly influenced the disintegration index. The *p*‐value of <0.0001 showed that the model was significant and there was only 0.01% chance that this level of fit could occur due to noise. The normal probability plot of the studentized residuals validated the assumption of normally distributed errors with constant variance and zero mean. The plots of residuals against predicted values, factor levels, and run orders showed a virtually constant variance over the variable ranges (not shown). Therefore, all the plots and statistics were acceptable, and it could be concluded that the model was adequate to explain the behavior of the system in terms of disintegration index, explaining approximately 98% (due to *R* ^2^) of the variability of *Z*.

The following 2FI model was accordingly chosen for the disintegration index:

(10) $$Z=0.37-0.10A+0.024A^{2}-0.030B+0.033B^{2}-0.14C+0.056C^{2}-0.042AB+7.02710^{-3}A^{2}B+0.021AB^{2}-0.045A^{2}B^{2}-0.070AC+0.056A^{2}C+0.073AC^{2}-0.018A^{2}C^{2}+0.023BC-0.034B^{2}C+0.023BC^{2}-0.019B^{2}C^{2}$$

This function describes how the experimental variables and their interactions influence the disintegration index. All the main factors and interactions of the factors were statistically significant in determining *Z* with a 95% confidence level. Temperature (*C*) had the greatest effect on *Z*, followed by electric field intensity (*A*), field intensity–temperature interaction (*AC*), and (*AC* ^2^). The positive values of these effects revealed that the increase in these parameters increased *Z*. Conversely, negative values of the effects decreased the response (*Z*). The predicted *R*‐squared of 0.7801 was in reasonable agreement with the adjusted *R*‐squared of 0.9372. Adequate precision measures the signal‐to‐noise ratio. Because a ratio of >4 is desirable, the ratio of 21.697 indicates an adequate signal.

**Figure 2:**
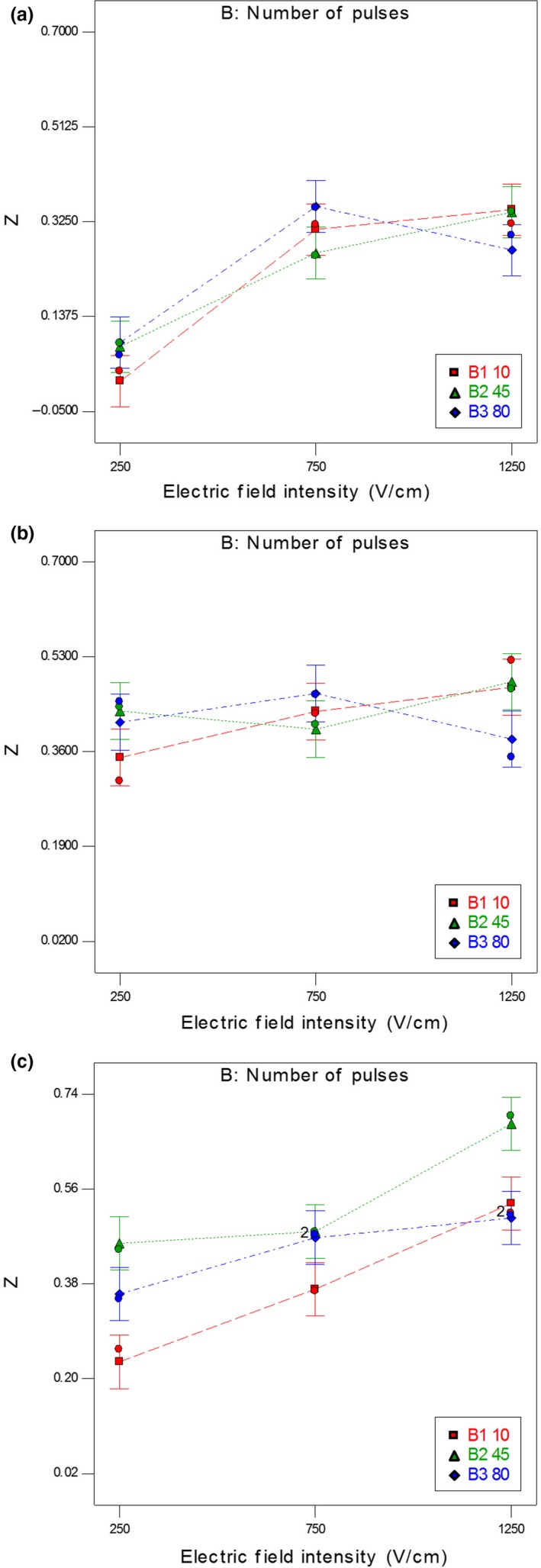
The interaction effect of electric field intensity and number of pulses on the disintegration index at (a) *T* = 20°C, (b) *T* = 45°C, and (c) *T* = 70°C after 255 min of thermal treatment

Figure 2(a‐c) shows the interaction effect of electric field intensity and the number of pulses on the disintegration index at 20, 45, and 70°C after 255 min of thermal treatment. At 45 pulses, cell membrane degradation was more evident. By increasing the number of pulses to higher than 45, no significant increase was observed in the cell disintegration index. This effect was resulted from the breakdown of most carrot cell membranes at 45 pulses. The results of other studies have also suggested that the disintegration index was increased with increasing the number of pulses up to a maximum value and, then, decreased. For example, in the sugar beet tissue, the maximum breakdown was achieved after 60 pulses, and excess pulsations were unnecessary (Maskooki & Eshtiaghi, 2012). The mechanism of this phenomenon has been described in other studies (Sale & Hamilton, 1967; Zimmermann, 1986). Longer pulses are more effective, and their effect is more obvious at the room temperature and moderate electric fields (De Vito, Ferrari, Lebovka, Shynkaryk, & Vorobiev, 2008)

By increasing the number of pulses in the range of 45–80 at 1,250 V/cm, the disintegration index *Z* first increased and, then, decreased, showing a limit for the increase in the values for electric fields. Bazhal, Lebovka, and Vorobiev (2003) investigated the effect of electric field intensity on the maximal disintegration index of vegetable tissues (Bazhal et al., 2003). With increasing the electric field and, subsequently, the energy input, the specific disintegration factor passed through a maximum point. Increasing the electric field strength was necessary for the membranes' damage because the secondary cell walls increased resistance in the membrane area. At 250 and 750 V/cm, no significant difference was observed in the disintegration index by increasing the number of pulses from 45 to 80.

**Figure 3:**
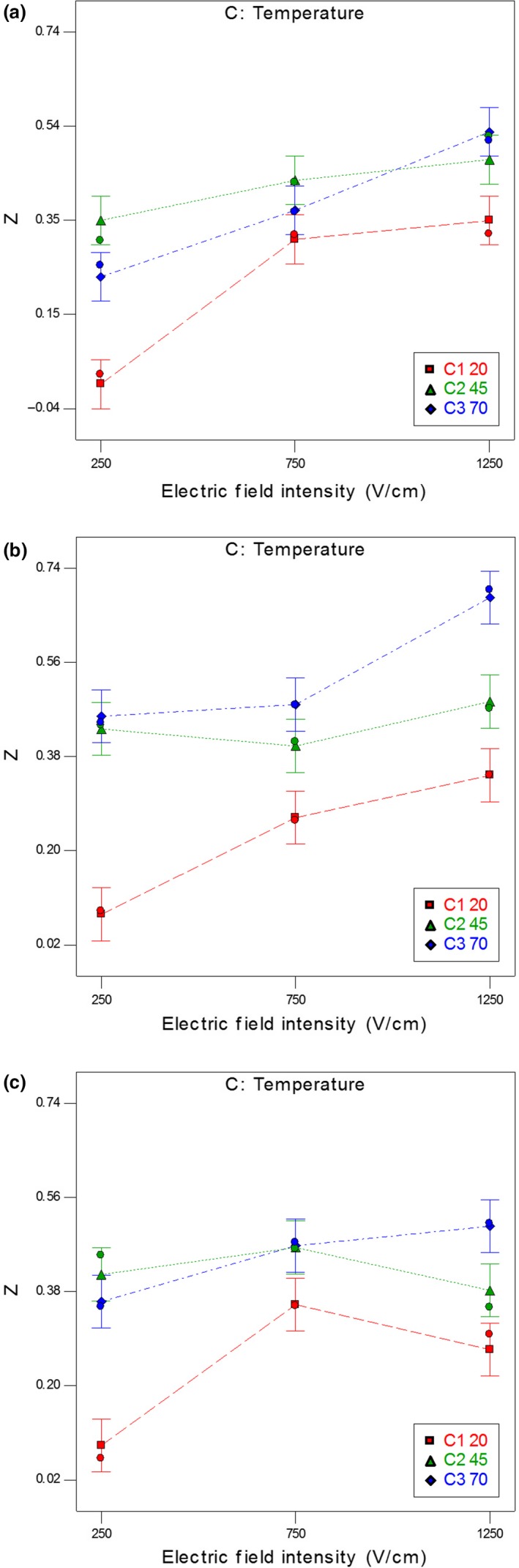
The interaction effect of electric field intensity and temperature on the disintegration index at (a) *n* = 10, (b) *n* = 45, and (c) *n* = 80 pulses after 255 min of thermal treatment

Figure 3(a–c) manifests the interaction effects of electric field intensity and temperature on the carrot tissue's disintegration index at 10, 45, and 80 pulses after 255 min of thermal treatment. As can be seen, increasing temperature and electric field intensity at a higher level (1,250 V/cm and 70°C) resulted in the significant increase of the disintegration index. However, at lower levels of the electric field (250 V/cm), the disintegration index was decreased by increasing the temperature to higher than 45°C. According to Lebovka, Shynkaryk, El‐Belghiti, et al. (2007), this effect can be related to the generation of small gas bubbles that screen the electrodes at high temperatures (Lebovka Shynkaryk, & Vorobiev, 2007b, 2007c).

Based on Lebovka et al. (2005), PEF treatment under moderate heating (<50°C) can help achieve a high degree of tissue damage at a moderate electric field (Lebovka et al., 2005). The enhancement of field intensity resulted in high degree of tissue damage. The complex dependences of disintegration index Z on electric field intensity E and temperature T are due to the existence of a wide range of cells with different geometries and sizes (Lebovka Shynkaryk, & Vorobiev, 2007b, 2007c).

From Figure 3, by increasing the electric field intensity to over 750 V/cm at moderate temperatures in the range of 20–45°C, the increase in the disintegration index was not significant at the 95% confidence level. These results were in agreement with those of other studies conducted on the apple tissue (Bazhal et al., 2003). With increasing the electric field of 1,200 to 1,500 V/cm in the apple tissue, no significant difference was observed in the disintegration index. According to Bazhal et al. (2003), disintegration index *Z* in small quantities of *E* (*E* < 300–500) has one or more saturation levels and may stabilize after an initial increase. If the treatment is continued, a further increase in σ and, subsequently, *Z* is observed after a long time. At higher values of *E*, *Z* has only one saturation level and reaches the saturation level after a short time.

**Figure 4:**
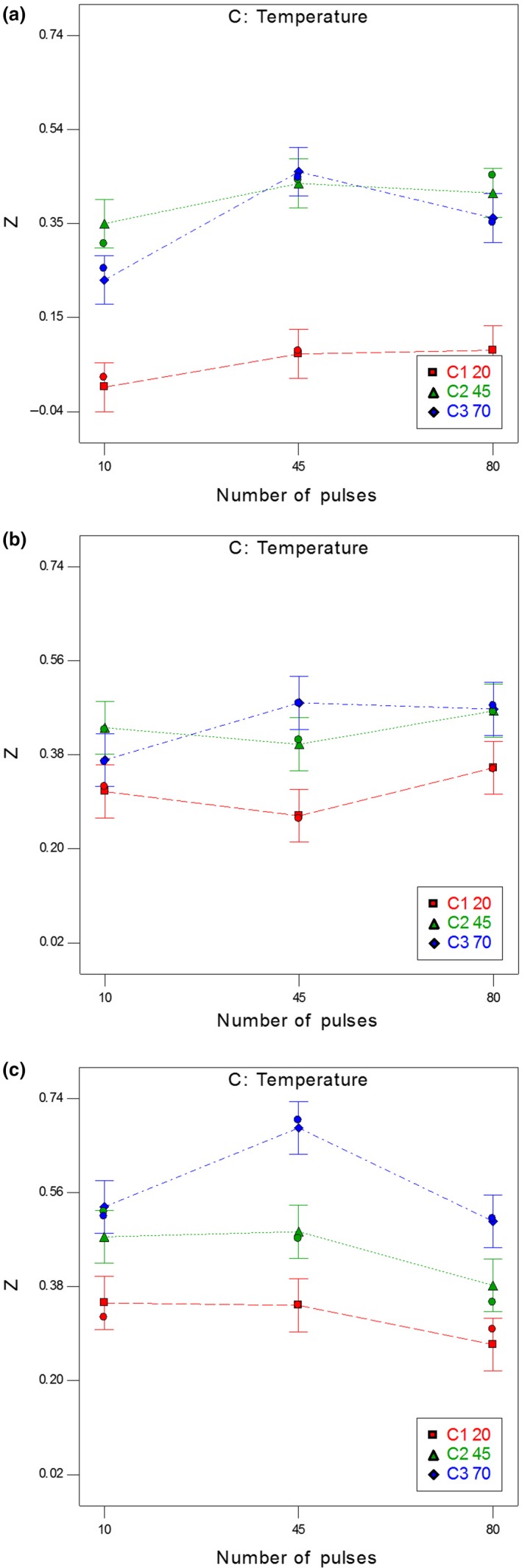
The interaction effect of the number of pulses and temperature on the disintegration index at (a) 250 V/cm, (b) 750 V/cm, and (c) 1,250 V/cm after 255 min of thermal treatment

Figure 4(a‐c) depicts the interaction effects of the number of pulses and temperature on the disintegration index. By increasing the temperature to over 45°C at 10 and 80 pulses, no significant difference was observed in disintegration index *Z* at the 95% confidence level. This effect, as already mentioned, was because *Z* reached the saturation level.

According to Figures 2 and 4, the disintegration index increased by increasing the number of pulses up to 45 pulses at the electric field intensity of 250 V/cm. In the higher electric field (>250 V/cm), there was no significant difference in the disintegration index by increasing the number of pulses. According to Bazhal et al. (2003), the optimal electric field *E*_opt_ for cells with a secondary cell wall in carrot, potato, cucumber, and apple is equal to 200–400 V/cm (Bazhal et al., 2003). Consequently, the electric field intensity of 750 V/cm and above as well as increasing the number of pulses and, subsequently, the treatment time did not create any significant effects on disintegration index *Z*.

### 3.4 Process optimization

After analyzing the effects of different variables and their interactions on the responses, it is important to find the best conditions to achieve an optimal response in the range of experiments. The experimental design software has the ability to find the best response. As mentioned before, the FFD model was used for optimization. Table 2 shows the experiments used in this method. The optimization of the responses was performed by a multiple‐response method, known as desirability (*D*) function, to optimize different combinations of process variables such as field intensity (*E*), the number of pulses (*n*), and temperature (*T*). This process aimed to improve the conditions to increase the yield of solute (c*) and disintegration index (*Z*) by considering minimum energy consumption that was hidden in all of the three factors. To achieve maximum desirability, the three factors were set within the range. Then, c* and *Z* were set to maximum.

As already mentioned, in the electric fields of higher than 300–400 V/cm in carrot tissue, 10 pulses (treatment time = 10 s) were enough to achieve maximum yield extraction. The yield was increased by increasing the electric field up to 750 V/cm. Afterward, no significant difference was found. However, by increasing the number of pulses to more than 10, no significant difference was seen in c*. Increasing the field intensity alternatively resulted in larger amounts of *Z* at 10 pulses. However, when the temperature was higher than 45°C, no significant difference was observed in *Z*. According to the two observed situations, the optimal conditions for both of the responses simultaneously included the electric field intensity of 750 V/cm, pulse number of 10, and temperature of 45°C. In this case, the solute yield extraction and the disintegration index were 64% and 0.43, respectively.

## 4 CONCLUSION

In this study, the method of pulsed electric field was utilized for sugar extraction from carrot. Design–Expert software and FFD were employed for obtaining the interaction of factors (electric field intensity, number of pulses, and temperature) and to find optimal conditions. The sugar extraction yield and disintegration index were measured as responses. The importance levels of electric field strength and temperature were almost identical based on the responses obtained. As a result, by the simultaneous application of a moderate electric field to the tissue at an average temperature, the same yield that was obtained at high temperatures can be obtained without any electric field, thereby saving energy consumption. The optimal conditions for the extraction process including the highest extraction yield and disintegration index can also be achieved under these situations.

## CONFLICT OF INTEREST

None declared.

## ETHICAL APPROVAL

This study was approved by Department of Chemical Engineering, Faculty of Engineering, Ferdowsi University of Mashhad, Iran.
